# A simple and efficient attack on the Merkle-Hellman knapsack cryptosystem

**DOI:** 10.1371/journal.pone.0322726

**Published:** 2025-05-28

**Authors:** Jingguo Bi, Lei Su, Haipeng Peng, Lin Wang

**Affiliations:** 1 School of Cyberspace Security, Beijing University of Posts and Telecommunications, Beijing, China; 2 State Key Laboratory of Cryptology, Beijing, China; 3 National Key Laboratory of Security Communication, Chengdu, China; Deakin University, AUSTRALIA

## Abstract

The Merkle-Hellman knapsack cryptosystem was one of the two earliest public key cryptosystems, which was invented by Merkle and Hellman in 1978. One can recover the equivalent keys by using Shamir’s method. The most time-consuming part of Shamir’s attack is to recover the critical intermediate parameters by solving an integer programming problem with a fixed number of variables, whose worst-case complexity is exponential of the number of variables. In this paper, we present an improved algorithm to analyze the basic Merkle-Hellman public key cryptosystem. The main idea is to recover a partial super-increasing sequence as equivalent private key, which is the main difference from Shamir’s. More precisely, we first obtain a super-increasing sequence by invoking the LLL algorithm on a special lattice with a small dimension. We can recover most part of the plaintext from the tail by solving the super-increasing knapsack problem. Finally, we get the first part of plaintext by solving a size-reduced knapsack problem. Experimental data shows that one can recover the whole plaintext in less than 1 second on a laptop for the typical parameters of the Merkle-Hellman cryptosystem, whose time complexity is reduced by a polynomial level compared with Shamir’s algorithm.

## 1 Introduction

Merkle-Hellman cryptosystem, which is a kind of public key cryptosystem, was proposed by Merkle and Hellman in 1978 [1]. Its security was based on the hardness of the knapsack problem. This scheme used super-increasing sequences and modular multiplication to construct a trapdoor, making it easy to decrypt with the private key. Attacks on this cryptographic system can be divided into two main categories: one is to directly solve the knapsack problem, and the other is to find equivalent keys.

Many algorithms have been proposed to solve the knapsack problem. In 1985, Lagarias and Odlyzko proposed a direct way to use lattices to solve the subset sum problem [2]. This method does not rely on any properties of the subset sum instance and only works when the density is sufficiently small. In 1991, Coster, Joux, LaMacchia, Odlyzko, Schnorr and Stern [3]made a small adjustment to the original method, raising the upper limit of applicable density from 0.6463 to 0.9408. However, there are no efficient lattice-based methods for solving the knapsack problem with density close to 1. For this case, the state-of-the-art algorithm is proposed by Schroeppel and Shamir [4], which runs in O(n · 2^*n*/2^) time and uses O(n · 2^*n*/4^) bits of memory. This algorithm runs in the same time as the basic birthday-based algorithm for knapsack problem introduced by Horowitz and Sahni [5], but has much lower memory requirements. In 2010, Howgrave-Graham and Joux proposed two new algorithms that improve this bound [6], reducing the running time further with reasonable heuristics. Besides, intelligent algorithms such as genetic algorithm [7, 8] and ant colony algorithm [9] are also used to crack the Merkle-Hellman cryptosystem.

Another way to break the Merkle-Hellman cryptosystem is to solve the private key. In 1982, Shamir proposed an algorithm to break it in polynomial time [10]. Shamir noticed an "unusual" relationship between parameters, whose essence is that modular multiplication cannot perfectly hide the information of the private key. In 2019, Liu and Bi proposed an attack based on lattice [11]. They use orthogonal lattice technique as the main tool and obtain a $\text{O}({\text{n}}^{7})$ speed-up compared with Shamir’s algorithm.

Currently, National Institute of Standards and Technology (NIST) is collecting post-quantum algorithms from all over the world, and knapsack ciphers are a promising class of candidate algorithms. The hardness of the knapsack problem is NP-complete and it can resist the attack of a quantum computer. Some improved knapsack algorithms have been proposed in recent years [12–15]. In this paper, we propose an improved method based on Shamir’s attack for cracking the Merkle-Hellman cryptosystem, which is helpful to the cryptanalysis of knapsack-based schemes.

### 1.1 Contributions

In this paper, we contribute to the body of knowledge on lattice-based attacks on the Merkle-Hellman cryptosystem by presenting an improved algorithm. Our approach differs from previous works in that we focus on recovering a partial super-increasing sequence as the equivalent private key, which is a novel way of utilizing the lattice reduction results. By invoking the LLL algorithm on a specially constructed small-dimensional lattice, we are able to bypass the most time-consuming integer programming step in Shamir’s attack, thereby achieving a significant reduction in time complexity.

Let *n* denote the number of the public key elements of the Merkle-Hellman cryptosystem and *L* is the length of input. *l* is the number of variables we use. In our improved method, the most time-consuming part is finding equivalent $M^{\prime }$ and $U^{\prime }$ by invoking the LLL algorithm [16]. Its complexity depends on the LLL algorithm, which is $\text{O}({\text{l}}^{6}{\text{L}}^{3})$ for classical LLL and can be further reduced using ${\text{L}}^{2}$ algorithm [17]. In our attack, we choose *l* = 20. Note that L=O(n) and l=O(1), the time complexity of our attack is $\text{O}({\text{n}}^{3})$, and the complexity of Shamir’s attack is $O(n^{15}n\log n)$, which means we get a $\text{O}({\text{n}}^{13})$ speed-up compared with Shamir’s algorithm.

Our work not only advances the understanding of how lattice theory can be applied to break the Merkle-Hellman cryptosystem but also provides new insights into the potential weaknesses of similar knapsack-based cryptographic schemes. The experimental results that demonstrate the efficiency of our attack further highlight the importance of considering lattice-based attacks when evaluating the security of cryptographic algorithms, especially in the context of post-quantum cryptography where the relevance of such attacks is expected to grow.

### 1.2 Organization

We organize the paper as follows. In Section 2, we recall the background on lattice theory, the Merkle-Hellman cryptosystem and Shamir’s attack algorithm. Section 3 shows our improved method that recovers the equivalent keys. Section 4 provides the experimental data of this method. Finally, we conclude the paper in Section 5.

## 2 Preliminary

### 2.1 Lattice

There exist *n* linearly independent vectors $\text{B}=\{{\text{b}}_{1},{\text{b}}_{2},...,{\text{b}}_{n}\}\subset {\mathbb{R}}^{m}$. A lattice generated by B is the set of all the integer linear combinations of the basis vectors.


$$ℒ(\text{B})=\{\sum x_{i}{\text{b}}_{i}:x_{i}\in \mathbb{Z}\}$$

A classic problem related to lattice is SVP (shortest vector problem): find a non-zero vector with the shortest Euclidean norm in lattice. The length of the shortest non-zero vector in lattice ℒ is denoted by $\lambda_{1}(ℒ)$.

**Definition 1** (SVP). *Given a basis of a lattice* ℒ, *find a non-zero vector* 𝐮∈ℒ, *such that* ∥𝐮∥≤∥𝐯∥ *for any vector* 𝐯∈ℒ⧵{0}.

The length of the shortest vector in the lattice is denoted by $\lambda_{1}$, and the Gaussian heuristic can be used to estimate its value. Gaussian heuristic says that in a random lattice, the length of the shortest vector is approximately:


$$\lambda_{1}\approx \frac{{\Gamma \left(n/2+1\right)}^{1/n}}{\sqrt{\pi }}\cdot {\left(\det\left(ℒ\right)\right)}^{1/n}\approx \sqrt{\frac{n}{2\pi e}}\cdot {\left(\det\left(ℒ\right)\right)}^{1/n}$$

where Γ(x) is the gamma function.

Given the basis B of lattice ℒ, the LLL algorithm [16] can output a set of short vectors in polynomial time, which is called LLL-reduced basis. Let $\left({𝐛}_{1},\ldots ,{𝐛}_{n}\right)$ be an LLL-reduced basis of a lattice ℒ. Then:

1. $\|{𝐛}_{1}\|\leq 2^{\frac{n-1}{4}}(det(ℒ)){\;}^{\frac{1}{n}}$.
2. $\left\|{𝐛}_{1}\|\leq 2^{\frac{n-1}{2}}\lambda_{1}(ℒ\right)$.

### 2.2 Knapsack problem

**Definition 2** (Knapsack Problem). *Given a set of non-negative integers $a_{1},a_{2},\cdots ,a_{n}$, and a value sum s from a subset of the whole set, determine the subset to sum s.*

Formally, the knapsack problem is given a set of non-negative integers $a_{1},a_{2},\cdots ,a_{n}$ and finds $x_{1},x_{2},\cdots ,x_{n}\in \{0,1\}$ such that $\sum_{i=1}^{n}a_{i}x_{i}=s$.

The knapsack problem is a well-known NP-complete problem. In recent years, new algorithms have been proposed to solve this problem, whose time complexity and the space complexity are all exponential of n [6, 18, 19]. However, there is one kind of easy knapsack problem we define below.

**Definition 3** (Super-increasing Knapsack Problem). Given a set of non-negative integers $a_{1},a_{2},\cdots ,a_{n}$, and a value sum s from a subset of the whole set, where $a_{i}>\sum_{j=1}^{i-1}a_{j}$, determine the subset to sum s.

It is easy to solve a super-increasing knapsack. Simply take the total weight of the knapsack s and compare it with the largest weight *a*_*n*_ in the sequence. If *s*<*a*_*n*_, then it is not in the knapsack, i.e.*x*_*n*_ = 0, otherwise, *x*_*n*_ = 1. Subtract *a*_*n*_ from the total s, and compare with the next highest number. Keep working this way until the total reaches zero.

### 2.3 Basic Merkle-Hellman cryptosystem

Here we have a brief introduction to the basic Merkle-Hellman cryptosystem. Alice wants to send a message *m* to Bob by utilizing the Merkle-Hellman cryptosystem.

**Key generation**:

Bob selects a super-increasing sequence $\text{b}=\{b_{1},b_{2},...,b_{n}\}$, which satisfies $b_{i}>\sum_{j=1}^{i-1}b_{j}$ for any i∈(1,n]. Chooses integer *M*,*W* s.t. $M>\sum_{i=1}^{n}b_{i}$ and gcd(M,W)=1. Calculate


$$a_{i}\equiv b_{i}W(mod\;M).$$

Then Bob’s public key as $\text{a}=(a_{1},a_{2},...,a_{n})$, and Bob’s private key is (b,W,M).

**Encryption**:

Alice wants to send the plaintext message $\text{m}=(x_{1},x_{2},...,x_{n})\in \{0,1{\}}^{n}$ to Bob. Alice inquires about Bob’s public key and then encrypts the message *m* as follows.


$$c=\sum_{i=1}^{n}x_{i}a_{i}.$$

The ciphertext is *c*.

**Decryption**:

Bob receives the ciphertext *c*, calculates $c^{\prime }=cW^{-1}(mod\;M)$.

Note that


$$c^{\prime }=cW^{-1}(mod\;M)=\sum_{i=1}^{n}x_{i}a_{i}W^{-1}(mod\;M)=\sum_{i=1}^{n}x_{i}b_{i}(mod\;M).$$

Because that $M>\sum_{i=1}^{n}b_{i}$, then one have $c^{\prime }=\sum_{i=1}^{n}x_{i}b_{i}$.

Bob can recover the plaintext message *m* by solving the super-increasing subset sum problem, which is easy.

### 2.4 Shamir’s attack

The core idea of the algorithm is to discover the "unusual" relationship between the parameters. Let us first consider the size of each parameter in the cryptosystem. *a*_*i*_ is a *dn* bit number and typical values of *d* and *n* are 2 and 100, respectively, so *a*_*i*_ is generally 200 bits. *M*’s size is 200 bits and *b*_*i*_ is chosen as *dn*−*n* + *i*−1, which is *n* + *i*−1 bits in general situations.

Note that $U=W^{-1}(mod\;M)$, so the size of *U* is also 200 bits. From $b_{i}=a_{i}U(mod\;M)$, then there is a positive integer *k*_*i*_ such that

$$b_{i}=a_{i}U-k_{i}M$$ (1)

Compared with *a*_*i*_*U*, the left side of the equation is a relatively small number, indicating that *a*_*i*_*U* and *k*_*i*_*M* are of the same order of magnitude, so *k*_*i*_ is also 200 bits. It can be obtained by Eq 1:


$$|\frac{U}{M}-\frac{k_{i}}{a_{i}|}=\frac{b_{i}}{Ma_{i}}\leq \frac{2^{n+i-1}}{2^{4n}}=2^{-3n+i-1}$$

We denote the number of *a*_*i*_ we use by *l*, for 1<*i*<*l*, we have


$$|\frac{k_{i}}{a_{i}}-\frac{k_{1}}{a_{1}|}\leq 2^{-3n+i-1}+2^{-3n}\leq 2^{-3n+l}$$

This indicates that

$$|k_{i}a_{1}-k_{1}a_{i}|\leq 2^{n+l}$$ (2)

We can observe from Eq 2 that the difference between two 4n bit numbers is a less than 2n bit number, which is a very unusual thing. Shamir pointed out that when *l*>*d* + 1, the integer programming problem can be used to find several sets of solutions in polynomial time. Once we get the value of *k*_1_, we can try to construct $\left(W^{\prime },M^{\prime }\right)$ pairs such that


$$b_{i}^{\prime }=a_{i}W^{\prime -1}\;(mod\;M^{\prime })\;,\;0<b_{i}^{\prime }<M^{\prime }\;,1\leq i\leq n$$

become a super-increasing sequence, thereby cracking the Merkle-Hellman cryptosystem. The time complexity of this attack is *O*(*n*^*l* + 10^*LlogL*) [20], where *L* is the length of the input.

## 3 Cryptanalysis

### 3.1 Observation of short vectors

Note that $a_{i}=W*b_{i}\;\mathrm{mod}\;M,i=1,\ldots ,n$, we have $b_{i}=W^{-1}*a_{i}\;\mathrm{mod}\;M,i=1,\ldots ,n$. Let $k_{i}\in \mathbb{Z}$ be the quotient such that $b_{i}=W^{-1}a_{i}+Mk_{i}$. The equations imply that

$$\frac{b_{1}a_{2}-b_{2}a_{1}}{M}=k_{1}a_{2}-k_{2}a_{1}$$ (3)

$$\frac{b_{1}a_{i}-b_{i}a_{1}}{M}=k_{1}a_{i}-k_{i}a_{1},3\leq i\leq n.$$ (4)

Take *n* = 100. Consider the bit length of the first few integers of the sequence *S*_*i*_, which is less than 99+i, it is far less than the bit length of *a*_*i*_, which is about 200. We try to recover the first *l*–1 integers $\frac{b_{1}a_{i}-b_{i}a_{1}}{M},i=2,\ldots ,l$. By the right part of the above equations, we define $h_{1}=(-a_{1},\ldots ,0),h_{2}=(0,-a_{1},\ldots ,0),\ldots ,h_{l-1}=(0,\ldots ,-a_{1})$, $h_{l}=(a_{2},\ldots ,a_{l})$. Then we apply LLL algorithm on the lattice $ℒ(h_{1},\ldots ,h_{l})$.

Define the following matrix:


$$H=\left[\begin{array}{cccc} -a_{1} & 0 & \cdots & 0\\ 0 & -a_{1} & \cdots & 0\\ \vdots & \vdots & \ddots & \vdots \\ 0 & 0 & \cdots & -a_{1}\\ a_{2} & a_{3} & \cdots & a_{l} \end{array}\right]$$

Obviously, the rank of lattice ℒ is *l*–1, and $h_{1},\ldots ,h_{l}$ is not a basis of the lattice. Therefore, the first short vector of the output of LLL algorithm is a zero vector. Besides, the norm of vector


$$\left(\frac{b_{1}a_{2}-b_{2}a_{1}}{M},\frac{b_{1}a_{3}-b_{3}a_{1}}{M},\cdots ,\frac{b_{1}a_{l}-b_{l}a_{1}}{M}\right)$$

in lattice ℒ is unusually small according to Eq 2 and Eq 4. It is the second shortest vector in the output, and the other *l*–2 vectors are much longer than the second one. The experiment data confirms our assumption.

Define

$$S_{i}=a_{i}k_{1}-k_{i}a_{1}$$ (5)

From Eq 3 and Eq 4, we have


$$\left(k_{2},k_{3},...,k_{l},k_{1})H=(S_{2},S_{3},...,S_{l}\right)$$

then the vector $\left(S_{2},S_{3},...,S_{l}\right)$ is the shortest in lattice ℒ. Because that we choose *l* is small, sometimes choosing *l* = 10, the rank of lattice ℒ is *l*–1, one can recover $S_{i},2\leq i\leq l$ very quickly.

**Lemma 1.** *Let ℒ be the lattice generated by the basis matrix H. The target vector $𝐒=(S_{2},\ldots ,S_{l})$ defined above is the shortest vector in the lattice ℒ , where $S_{i}=a_{i}k_{1}-k_{i}a_{1}$,provided l > 3.*

*Proof:* The target vector $𝐒=(S_{2},\ldots ,S_{l})$ is defined as $S_{i}=a_{i}k_{1}-k_{i}a_{1}$ for 2≤i≤l, where $|S_{i}|\leq 2^{n+l}$ by Eq. (2). Its Euclidean norm satisfies:


$$\left\|𝐒\|\leq \sqrt{l-1}\cdot 2^{n+l}=O(2^{n+l}\right)$$

According to the lattice basis *H*, the determinant of lattice ℒ is calculated as:


$$\det(ℒ)=a_{1}^{l-2}\approx {\left(2^{2n}\right)}^{l-2}=2^{2n(l-2)}$$

By the Gaussian heuristic, the expected shortest vector length in lattice ℒ is:


$$\lambda_{1}(ℒ)\approx \sqrt{\frac{l-1}{2\pi e}}\cdot \det(ℒ{)}^{1/(l-1)}=O\left(2^{\frac{2n(l-2)}{l-1}}\right)$$

Obviously, when *l*>3, $2^{n+l}<2^{\frac{2n(l-2)}{l-1}}$, and the ratio of the Gaussian heuristic-predicted shortest vector length to the target vector length exhibits a monotonic increase with growing parameter *l*, demonstrating asymptotic divergence behavior.

Therefore, the target vector is the shortest vector in the lattice ℒ provided *l* > 3. ◻

**Remark 1.** *Note that the LLL algorithm guarantees that the first reduced basis vector ${𝐛}_{1}$ satisfies:$\|{𝐛}_{1}\|\leq 2^{(l-2)/4}$ · $\det{\left(ℒ\right)}^{1/(l-1)}=O\left(2^{2n}\right)$. It is well-known that the LLL algorithm performs well when the dimension of the lattice is smaller than 50. From Lemma 1, one can recover the target vector* **S** *by utilizing LLL algorithm when l is not big. The gap between the length of the target vector and the expected length of Gaussian heuristic becomes larger, then LLL algorithm will recover the target vector much more easily. It is validated by the experimental data.*

Our approach shares similarities with the one presented in [21], yet it differs in two aspects. Firstly, our lattice has a lower dimension. Secondly, we offer a more rigorous theoretical analysis ensuring the lattice’s ability to find the target vector.

### 3.2 Recover the equivalent key

We all know that one can solve the super-increasing knapsack problem in *n* times arithmetic operations. Shamir’s method aims to recover the whole group of the equivalent keys, and then recover the plaintext based on the equivalent keys. In this paper, we propose a method to recover the whole group of equivalent keys except the first small part of the equivalent keys, and then, we can recover most of the plaintext from the tail. After that, one can easily search for the remaining part of the plaintext, because the size of the knapsack problem is quite small.

From the last subsection, one can obtain $S_{2},...,S_{l}$ and $k_{1}^{\prime },k_{2}^{\prime },...,k_{l}^{\prime }$ by using the LLL lattice reduction algorithm. Notice that $k_{i}^{\prime }$ may be equal to the original *k*_*i*_ or not, but as long as it satisfies the relationship $S_{i}=a_{i}k_{1}^{\prime }-k_{i}^{\prime }a_{1}$, it will not affect our successful cracking of the cryptosystem. For convenience, we will not distinguish *k*_*i*_ and $k_{i}^{\prime }$ in the following, and they will all be recorded as *k*_*i*_.

Interestingly, we observe that there exists a fixed integer *i*_0_, the sequence $S_{i_{0}},S_{i_{0}+1},...,S_{n}$ forms a super-increasing sequence, which is an important property of the equivalent key we are looking for. Therefore, we regard *S*_*i*_ as the private key *b*_*i*_. The relationship between *b*_*i*_ and *a*_*i*_ is Eq 1. For *S*_*i*_ and *a*_*i*_, we can find an equation (5) with the same structure . Therefore, we obtain the equivalent key:


$${\text{b}}^{\prime }=(S_{1},S_{2},\cdots ,S_{i_{0}},\cdots ,S_{n})$$

$$U^{\prime }=k_{1}$$

$$M^{\prime }=a_{1}$$

**Lemma 2.** *In the parameters defined above, there exists an integer *i*_*0*_ *to be determined, such that* $S_{i_{0}},S_{i_{0}+1},...,S_{n}$ forms a super-increasing sequence.*

*Proof:* It can be derived from Eq 1 (5) that


$$MS_{i}=b_{i}a_{1}-b_{1}a_{i}$$

As long as it is proved that *MS*_*i*_ is a super-increasing sequence, we can get that *S*_*i*_ is also a super-increasing sequence. Let’s calculate that

$$MS_{i+1}-\sum_{k=1}^{i}MS_{k}=b_{i+1}a_{1}-b_{1}a_{i+1}-\sum_{k=1}^{i}(b_{k}a_{1}-b_{1}a_{k})\;=a_{1}(b_{i+1}-\sum_{k=1}^{i}b_{k})+b_{1}(\sum_{k=1}^{i}a_{k}-a_{i+1})$$ (6)

Note that *b*_*i*_ forms a super-increasing sequence, so $a_{1}(b_{i+1}-\sum_{k=1}^{i}b_{k})>0$. For the second term, there exists an integer *i*_0_, such that for any *i*>*i*_0_,

$$\sum_{k=1}^{i}a_{k}>a_{i+1}.$$ (7)

In the following we will prove that when i≥10, Eq 7 is true with a probability close to 1.

Consider a sequence of *i* independent random variables $a_{1},a_{2},\ldots ,a_{i}$, where each *a*_*k*_ (k=1,2,…,i) represents a random integer uniformly chosen from the discrete interval [0,M−1]. Let $R_{i}=a_{1}+a_{2}+\cdots +a_{i}$ denote the sum of these *i* random integers. Each *a*_*k*_ is a discrete random variable, and its expectation and variance are derived from the properties of a uniform distribution. Specifically, the expectation of *a*_*k*_ is given by:


$$E(a_{k})=\frac{M-1}{2},$$

which is the mean of the uniform distribution over [0,M−1]. The variance of *a*_*k*_ is:


$$\text{Var}(a_{k})=\frac{(M-1)M}{12}.$$

Since *R*_*i*_ is the sum of *i* independent and identically distributed random variables, the expectation and variance of *R*_*i*_ are:


$$E(R_{i})=i\cdot E(a_{k})=i\cdot \frac{M-1}{2},$$

and


$$\text{Var}(R_{i})=i\cdot \text{Var}(a_{k})=i\cdot \frac{(M-1)M}{12}.$$

To compute *P*(*R*_*i*_>*M*), we use the Central Limit Theorem (CLT), which states that the sum of a sufficiently large number of independent and identically distributed random variables converges to a normal distribution. Consequently, *R*_*i*_ can be approximated as:


$$R_{i}\sim 𝒩(E(R_{i}),\text{Var}(R_{i})).$$

By standardizing *R*_*i*_ into the standard normal variable *Z*, we write:


$$Z=\frac{R_{i}-E(R_{i})}{\sqrt{\text{Var}(R_{i})}}.$$

The probability *P*(*R*_*i*_>*M*) is then expressed as:


$$P(R_{i}>M)=P\left(Z>\frac{M-E(R_{i})}{\sqrt{\text{Var}(R_{i})}}\right).$$

Substituting $E(R_{i})=i\cdot \frac{M-1}{2}$ and $\text{Var}(R_{i})=i\cdot \frac{(M-1)M}{12}$, the argument of the standard normal cumulative distribution function (CDF) becomes:


$$\frac{M-i\cdot \frac{M-1}{2}}{\sqrt{i\cdot \frac{(M-1)M}{12}}}.$$

Finally, the probability *P*(*R*_*i*_>*M*) is expressed as:


$$P(R_{i}>M)=1-\Phi \left(\frac{M-i\cdot \frac{M-1}{2}}{\sqrt{i\cdot \frac{(M-1)M}{12}}}\right),$$

where Φ(x) denotes the CDF of the standard normal distribution. This formulation provides the exact probability of the sum of the *i* random integers exceeding *M*, based on their uniform distribution over [0,M−1].

For the specific case where *i* = 10 and *M* is a 200-bit integer, substituting these values into the formula gives the standardized value:


$$Z=\frac{M-10\cdot \frac{M-1}{2}}{\sqrt{10\cdot \frac{(M-1)M}{12}}}.$$

Numerically approximating this for *M* = 2^200^, we find:


$$P(\sum_{k=1}^{i}a_{k}>a_{i+1})\geq P(R_{10}>M)\approx 0.999994.$$

It should be noted that the Central Limit Theorem is most accurate when the number of summands *i* is sufficiently large. For *i* = 10, the approximation is reasonable but not exact. Nonetheless, for practical purposes, the CLT-based approximation remains highly effective, particularly for large *M*, where the relative error becomes negligible.

In summary, the above analysis shows that when i≥10, $\sum_{k=1}^{i}a_{k}-a_{i+1}>0$ holds with a probability close to 1. ◻

**Remark 2.** *Here we use another method to give a lower bound on the probability that* Eq 7 *holds.If we can prove that $\sum_{k=1}^{i}a_{k}>M$, then $\sum_{k=1}^{i}a_{k}>a_{i+1}$ must be true. The necessary condition for $\sum_{k=1}^{i}a_{k}>M$ is that at least two of $a_{1},a_{2},...,a_{i}$ are greater than *M*/2. Since $a_{i}\equiv b_{i}W(mod\;M)$ , M is very large, a can be approximately regarded as uniformly randomly selected from [0,M–1]. Then it is easy to calculate*


$$P(\sum_{k=1}^{i}a_{k}>a_{i+1})\geq 1-\frac{i+1}{2^{i}}.$$

*According to the above analysis, this probability rises as i increases and when *i* = 10, the probability that* Eq 7 *being true is not less than 98.9%.*

Based on this parameters setting, one can recover the plaintext $\left(m_{i_{0}},m_{i_{0}+1},\cdots ,m_{n}\right)$. For the remaining unknown part $m_{1},m_{2},\cdots ,m_{i_{0}-1}$, we can solve a knapsack problem with size *i*_0_−1, i.e.


$$\sum_{i=1}^{i_{0}-1}a_{i}m_{i}=c-\sum_{i=i_{0}}^{n}a_{i}m_{i}$$

This size-reduced problem can be solved easily. Because *i*_0_ is usually not larger than 5 in the practical cryptanalysis, whose probability exceeds 96%. See the experimental section later for details.

It can be seen from Remark 2 that the probability lower bound corresponding to the constant value of *i*_0_ is independent of *n* and its size is *O*(1), so the complexity of exhaustively enumerating $m_{1},m_{2},\cdots ,m_{i_{0}-1}$ is also *O*(1). According to Lemma 2 and Remark 2, the theoretical probability of $i_{0}\leq 10$ is 99.9994%, and the probability lower bound of $i_{0}\leq 10$ and $i_{0}\leq 20$ is 98.9258% and 99.9938% respectively. In the experiment, the appropriate parameter setting can ensure that *i*_0_ is all within 10, so 10 can be regarded as the "actual effective upper bound" of *i*_0_. In Remark 2, the lower bound of the probability corresponding to *i*_0_ decreases exponentially with its increase. Therefore, the theoretical upper bound of *i*_0_ will be a small integer, and an exhaustive search of this scale can be easily performed.

Finally, we recover the whole plaintext of the basic Merkle-Hellman cryptosystem. The whole description of our attack is given in Algorithm 1 below.

**Algorithm 1. Our algorithm for breaking basic Merkle-Hellman cryptosystem.**

**Require**: Public key a and Ciphertext *c*.

**Ensure**: Message m.

1: Construct the lattice basis matrix *H* mentioned in Sect.3.1.

2: Invoke the LLL algorithm to obtain a set of reduced basis.

3: Select the last coefficient corresponding to the shortest

non-zero vector as *k*_1_.

4: Let *n* be the length of public key.

5: $U^{\prime }\leftarrow k_{1},M^{\prime }\leftarrow a_{1}$.



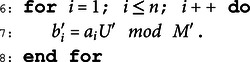



9: Determine *i*_0_, such that the sequence $b_{i_{0}},b_{i_{0}+1},...,b_{n}$ form a

super-increasing sequence.

10: $c^{\prime }=c\cdot U^{\prime }\;mod\;M^{\prime }$.

11: Obtain $m_{i_{0}},m_{i_{0}+1},\cdots ,m_{n}$ by solving the super-increasing

knapsack problem.

12: Solve the size-reduced knapsack problem

$\sum_{i=1}^{i_{0}-1}a_{i}m_{i}=c-\sum_{i=i_{0}}^{n}a_{i}m_{i}$ by enumeration the

  $m_{i},1\leq i\leq i_{0}-1$.

The most time-consuming part of our method is invoking the LLL algorithm to recover *k*_1_. The lattice basis matrix *H* is l×(l−1), and the complexity of invoking classical LLL algorithm on lattice *H* is $\text{O}({\text{l}}^{6}{\text{L}}^{3})$. Note that the value of *l* is a small integer and L=O(n), so the time complexity of this attack is $\text{O}({\text{n}}^{3})$.

BKZ algorithm [22] has stronger reduction capabilities than the LLL algorithm in practice. However, the dimension of our lattice is a small constant. The LLL algorithm can easily find the target vector from the lattice. The BKZ algorithm is a waste of talent here and can not bring any improvement. Therefore, we use the LLL algorithm instead of the BKZ algorithm in our method.

## 4 Experiments

According to Lemma 2, there exists an integer *i*_0_, such that the sequence $S_{i_{0}},S_{i_{0}+1}\mathrm{\backslash allowbreak}...,S_{n}$ forms a super-increasing sequence. Experiment data validates Lemma 2. We find that the first few elements of $S_{i},i<i_{0}$ are not always super-increasing. This will lead to incorrect decryption of the first few bits when solving this partially super-increasing knapsack problem directly. Fortunately, there is an upper limit for the number of error bits in decryption. When the size of plaintext is 100 bits, errors are usually concentrated in the first 5 bits in most cases, and rarely up to around 10 bits, which can be obtained by exhaustive methods by solving the size-reduced knapsack problem. Note that the *S*_1_ given by our algorithm is always 0, and the ciphertext obtained when the coefficient *x*_1_ is 0 and 1 in the subset sum problem is the same, so the first bit of plaintext can only be obtained by exhaustion.

In the experiment, we choose *n* = 100, l=10,20 and the size of the message is 100 bits. We define *T* as the number of message bits the algorithm successfully recovered. To check the effect of our method, we generate 1000 sets of public keys and ciphertext of Merkle-Hellman knapsack cryptosystem for each group of parameters. prop(T≥c) denotes the proportion of instances that successfully recover *c* bits.

Case I, we do not consider the final step of Algorithm.1, i.e. solving the size-reduced knapsack problem. The specific experiment data are shown in Table 1.When *n* = 100, experimental results show that the success rate of restoring 99 bits is stable at 84%, and the success rate of restoring 90 bits is up to 98.9% when *l* = 10. When *l* = 20, experimental data show that the success rate of restoring 99 bits is stable at over 85%, and the success rate of restoring 90 bits is up to 99.5%. It should be noted that when *T* = 99, we consider that all the plaintexts have been recovered, because the first plaintext can only be recovered by enumeration.

**Table 1:** Success rate of case I.

*n*	100		200		300	
*l*	10	20	10	20	10	20
prop(T≥90)	98.9%	99.5%	99.4%	100%	99.7%	99.8%
prop(T≥99)	84.0%	85.0%	82.4%	86.8%	83.7%	81.3%

Moreover, we examine the performance of the algorithm when *n* = 200 and *n* = 300 in Table 1. We find that when the scale of the system becomes larger, our method can still achieve excellent results. The probability of correctly recovering the plaintext is over 99%. As we have theoretically analyzed,the dimension of lattice *H* will not change as *n* increases, so the probability of successfully cracking the private key will not change significantly.

The conclusion we can draw is that the more public key used, the more plaintext bits *T* can be correctly recovered. However, *l* = 20 is enough. At this time, the failure probability of recovering 90% of the plaintext bits is negligible. The remaining plaintext of less than 10 bits can be obtained through exhaustive search.

Case II, we run the whole Algorithm 1. For the parameters *n* = 100, l=10,20, the size of the message is 100 bits, and the success rate is 100%. We can recover the plaintext for each set of parameters. Experimental data show that *i*_0_ is mostly concentrated in 1~5 when l=10,20. The value of *i*_0_ is shown in Table 2.

**Table 2:** Distribution of *i*_0_.

$i_{0}$	1	2	3	4	5	6	7	8	9	10	11~20	20+
*l* = 10	720	0	155	51	36	11	6	7	6	0	0	8
*l* = 20	741	0	153	49	30	16	6	5	0	0	0	0

We can see that in 1000 sets of data, more than 96% of *S* can form a super-increasing sequence after removing the first five digits at most no matter *l* is 10 or 20. The case of 20 +  indicates that the decryption failed. When *l* = 10, there is a probability of 8 out of 1000, and there is no failure when *l* = 20. Since the first element of *S* is 0, there is no case of *i*_0_ = 2. The experimental data also validates Lemma 2.

We used the same computing power to run Shamir’s algorithm and our algorithm. The running time of the two algorithms is shown in Table 3. As we can see, the running time of our algorithm is very short, which is much more efficient than Shamir’s. Moreover, as the size of *n* increases, the running efficiency of our algorithm is very little affected. This is because the lattice dimension used to solve the intermediate variables in our algorithm does not change with the increase of *n*. Accordingly, the increase of *n* has a very small impact on the complexity of lattice basis reduction, and the running time will not change significantly. Therefore, our algorithm can still perform well when *n* is large.

**Table 3:** Comparison of running time.

*n*	Running time of shamir’s algorithm(h)	Running time of our algorithm(s)
100	$5.84\times 10^{6}$	0.13
200	$1.01\times 10^{9}$	0.16
300	$1.72\times 10^{10}$	0.21

In summary, the more information of the public key we use, the higher the success rate should be. From Table 2, one can choose *l* = 20 conservatively for the typical parameters of the Merkle-Hellman cryptosystem. In this case, *i*_0_ will be smaller than 10 for the overwhelming probability, that one can recover the whole plaintext by solving a reduced knapsack problem with size smaller than 10.

## 5 Conclusion

Our paper presents a novel approach to cryptanalyzing the Merkle-Hellman Knapsack Cryptosystem, one of the earliest public-key cryptosystems. Our improvement lies in an enhanced algorithm that significantly reduces the time complexity for key recovery compared to Shamir’s method. By leveraging the LLL lattice reduction algorithm on a specially constructed lattice, we efficiently identify a partial super-increasing sequence, which serves as the equivalent private key. This approach allows for the rapid decryption of the plaintext message, with experimental data indicating a recovery time of less than 1 second on a standard laptop for typical parameters of the Merkle-Hellman cryptosystem.

The significance of this work mainly lies in two aspects: it not only provides a more efficient method for analyzing the security of Merkle-Hellman cryptosystem but also contributes to the broader field of cryptanalysis by offering insights into the potential vulnerabilities of knapsack-based cryptographic schemes. This could have implications for the ongoing development of post-quantum cryptographic algorithms, as the knapsack problem’s resistance to quantum attacks makes it a promising candidate for future secure communication protocols.

For future research, we propose several directions. First, it would be beneficial to explore the application of our method to other knapsack-based schemes and assess its generalizability. Besides, further optimization of the LLL algorithm for our specific use case could lead to even more significant performance gains. Lastly, the development of new cryptographic schemes that build upon the lessons learned from attacks like ours is essential to staying ahead in the ever-evolving field of cryptography.

## Supporting information

S1 File Main code. The experimental code of the algorithm in this paper and the environment configuration required to run the code.
(zip)
