# Supplementary material for: A simple and efficient attack on the Merkle-Hellman knapsack cryptosystem
Source: PLoS One. 2025 May 28;20(5):e0322726. doi: 10.1371/journal.pone.0322726 (PMC12118879; doi:10.1371/journal.pone.0322726)
Supplement: S1 File — The experimental code of the algorithm in this paper and the environment configuration required to run the code. (zip) [file pone.0322726.s001.zip › S1_File/Environment Configuration.pdf]

Requirement

- SageMath9.2

How to run

Run in jupyter notebook

```
%run <file path>/Attack Algorithm.py
```
